# The relationship between maternal health and neonatal low birth weight in Amman, Jordan: a case-control study

**DOI:** 10.25122/jml-2022-0257

**Published:** 2023-02

**Authors:** Amer Sindiani, Ekram Awadallah, Eman Alshdaifat, Shatha Melhem, Khalid Kheirallah

**Affiliations:** 1 Department of Obstetrics and Gynecology, Faculty of Medicine, Jordan University of Science and Technology, Irbid, Jordan; 2 Department of Public Health, Faculty of Medicine, Jordan University of Science and Technology, Irbid, Jordan; 3 Department of Obstetrics and Gynecology, Faculty of Medicine, Yarmouk University, Irbid, Jordan

**Keywords:** COVID-19, birth weight, neonate, pregnancy, preterm

## Abstract

This study aimed to examine the relationship between maternal health during pregnancy and low birth weight (LBW), as well as the impact of COVID-19 on the socio-economic status of pregnant women and its effect on LBW. The study was conducted in Amman, Jordan, and included 2260 mothers who visited Abu-Nusair comprehensive health center between January and December 2020. A matched case-control design was used with 72 cases and 148 controls selected for data collection through medical records and face-to-face interviews. Results showed that factors such as a monthly income of 400 JD or less, living with an extended family, exposure to passive smoking, maternal weight gain of 6–10 kg, maternal anemia, maternal hypertension, delivery by cesarean section, and previous history of LBW newborns were positively associated with an increased risk of LBW. Conversely, factors such as a monthly income above 700 JD, living with a core family, daily intake of iron, calcium, and vitamin D, prenatal visits, healthy food intake, and planning for pregnancy were associated with a lower risk of LBW. COVID-19 infection and its effects on work, family finances, antenatal care visits, and food supply were also positively linked with LBW. In conclusion, socioeconomic status, maternal health, COVID-19, and its impacts were significant risk factors for LBW.

## INTRODUCTION

Low birth weight (LBW) is a major public health burden due to its impacts on neonatal health, development, and survival [1]. Infants with LBW face a much higher risk of mortality, with rates 20 times higher compared to those with normal birth weight (NBW) [2]. Additionally, LBW newborns are at increased risk of experiencing cognitive deficits, metabolic diseases, motor delays, cerebral palsy, and other psychological and behavioral problems [1]. The World Health Organization defines low birth weight (LBW) as "live births weighing less than 2500 grams at birth, regardless of gestational age" [3]. LBW can also be defined as a birth weight below the 10^th^ (or 5^th^) percentile for gestational age or less than 2 standard deviations below the mean for gestational age [4]. Maternal nutrition, physical and psychological health, social status, and socioeconomic factors all play a role in determining the risk of LBW during pregnancy [5], with a higher prevalence in developing countries compared to developed countries.

Low birth weight is caused by a combination of factors, including intrauterine growth restriction (IUGR) and preterm birth. These conditions occur due to placental insufficiency, which impairs fetal nutrition and growth [1]. Maternal factors during pregnancy, such as nutrition, economic stability, and social factors, significantly impact neonatal weight [1]. The COVID-19 pandemic has exacerbated these challenges, leading to increased economic and social stress [6] and reducing access to adequate prenatal care, maternal follow-ups, and essential supplements, resulting in a higher incidence of LBW newborns during the lockdown period [7].

Globally in 2015, 14.56% of newborns had low birth weight, a condition that increases their risk of experiencing complications and mortality by 20 times compared to newborns with normal birth weight [1]. In Jordan, LBW is a significant public health concern as it is the leading cause of morbidity and mortality in newborns, with a prevalence rate of 13.8% in 2012 [8]. Therefore, this study aimed to assess the relationship between maternal health, obstetric outcomes, and LBW in Abu Nusair Comprehensive Center (ANCC) north of Amman between January and December 2020. In addition, we aimed to investigate the impact of COVID-19-related socioeconomic factors on neonatal low birth weight.

## MATERIAL AND METHODS

### Study design and setting

The study employed a matched case-control design in which controls were matched to cases based on age and social class (as indicated by the level of education of parents and employment) among women who visited Abu-Nusair comprehensive health center (ANCC) during the specified period. This study was conducted at ANCC, the second-largest comprehensive center in Amman and the biggest in the north of Amman.

The study population was dichotomized into two groups: cases and controls. The cases comprised mothers of term-singleton newborns with a birth weight of less than 2500 grams and without any congenital anomalies or deformities who visited the Abu-Nusair Comprehensive Health Center (ANCC) in 2020. The controls comprised mothers of term-singleton newborns with a birth weight ranging from 2500 grams to less than 4500 grams and without any congenital anomalies or deformities, who also visited the ANCC in 2020. Mothers with newborns with multiple births, a history of preterm deliveries, congenital anomalies, and any deformities were excluded from this study as those are common risk factors for LBW.

### Data collection

The initial sample size of all mothers eligible for the study was 2260. Data were obtained from medical files and during face-to-face interviews. Seventy-two cases were randomly selected from the total 166 cases using the last digit of the parent's mobile number, and 148 controls were chosen from 2094 controls based on matching age and social class (parents’ level of education, employment). Participants who failed to attend the interviews were excluded. The interview questionnaire consisted of four main sections: sociodemographic data, maternal health during pregnancy, obstetric outcomes, and COVID-19 infection and its associated socioeconomic impacts. The study method and protocol were approved by the Institutional Review Board (IRB) of Jordan University of Science and Technology and the Ministry of Health.

Demographic data included questions about age, academic level, working status, monthly family income, residency, and smoking status. Maternal health was assessed based on maternal weight gain during pregnancy, body mass index (BMI), number of prenatal care visits, multivitamin intake, dietary quality, sleep patterns, and medical conditions such as hypertension, diabetes, asthma, anemia, urinary tract infections, rheumatic diseases, systemic lupus erythematosus, and any other relevant conditions. We also assessed various factors related to obstetric outcomes, including parity, gender of the newborn, pregnancy planning, mode of delivery, history of miscarriages, type of pregnancy, gestational age at delivery, and previous LBW newborns.

**Figure 1:**
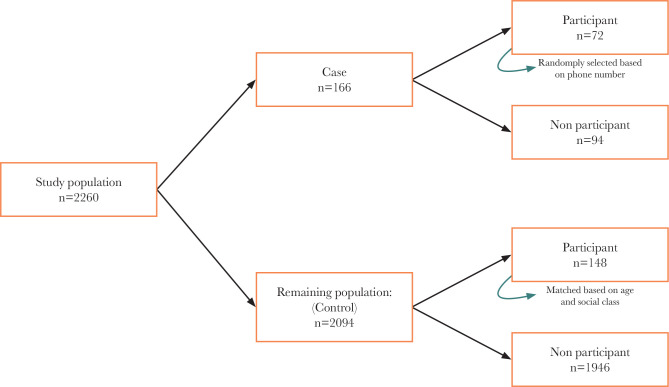
A schematic representation of the study sampling approach.

The last section of the interview focused on COVID-19 and included questions regarding infection status, hospital admission, job stability, food access, medication intake, antenatal care visits, and availability of multivitamins. Figure 1 shows a schematic summary of the sampling approach for this study.

### Statistical analysis

Data were analyzed using the Statistical Package of Social Science (SPSS) version 25 (IBM SPSS Statistics for Windows, Version 25.0. Armonk, NY: IBM Corp.). Frequencies and percentages were calculated for the categorical data, and a chi-square test (Fisher exact test) was conducted to compare the proportions of the categorical variables between two groups (case group and control group). Binary logistic regression analysis was performed to determine the associations between risk factors of LBW neonatal outcomes among the Jordanian women (case *vs*. control for each risk factor). The level of significance was set at p<0.05.

## RESULTS

### Socio-demographic characteristics

More than 65% of the case (66.7%) and control (66.2%) groups reported being unemployed. Around 23.6% of the participants had a monthly income of less than 400 JD, 47.3% had an income between 401–700 JD, and 29.1% had a monthly income exceeding 700 JD. A higher proportion of participants in the case group (51.4%) had a monthly income of 400 JD or less compared to the control group (10.1%), which was statistically significant (p<0.001). Conversely, a greater percentage of participants in the control group (35.1%) had a monthly income greater than 700 JD compared to the case group (16.7%), which was also statistically significant (p<0.001). A larger proportion of women in the case group lived with extended family members compared to the control group (36% *vs*. 20%) (p=0.001). In addition, a majority (75%) of the participants in the case group were exposed to passive smoking during pregnancy, compared to only 32.4% of the participants in the control group (p<0.001). These findings are summarized in Table 1.

**Table 1:** Socio-demographic and behavior factors (n=220).

Variables	Case (72) n (%)	Control (148) n (%)	Total	P-value
**Occupational status**				
Employed	24 (33.3)	50 (33.8)	74 (33.6)	0.947
Unemployed	48 (66.7)	98 (66.2)	146 (66.4)	
**Monthly income (JD)**				
≤400	37 (51.4)	15 (10.1)	52 (23.6)	**<0.001**
401–550	10 (13.9)	46 (31.1)	56 (25.5)	
550–700	13 (18.1)	35 (23.6)	48 (21.8)	
>700	12 (16.7)	52 (35.1)	64 (29.1)	
**Type of families**				
Core family	46 (63.9)	118 (79.7)	164 (74.5)	**0.001**
Extended family	26 (36.1)	30 (20.3)	56 (25.5)	
**Maternal cigarettes use**				
Yes	7 (9.7)	3 (2.0)	10 (4.5)	**0.010**
No	65 (90.3)	145 (98.0)	210 (95.5)	
**Maternal waterpipe use**				
Yes	12 (16.7)	17 (11.5)	29 (13.2)	0.287
No	60 (83.3)	131 (88.5)	191 (86.8)	
**Maternal smoking status**				
Cigarette only	2 (2.8)	1 (0.7)	3 (1.4)	0.084
Water pipe only	7 (9.7)	15 (10.1)	22 (10.0)	
Cigarette and water pipe	5 (6.9)	2 (1.4)	7 (3.2)	
Non-smoker	58 (80.6)	130 (87.8)	188 (85.5)	
**Passive smoking**				
Yes	54 (75.0)	48 (32.4)	102 (46.4)	**<0.001**
No	18 (25.0)	100 (67.6)	118 (53.6)	
Body Mass Index (BMI)*				
Underweight	3 (4.2)	7 (4.8)	10 (4.6)	0.753
Normal weight	52 (73.2)	97 (66.0)	149 (68.3)	
Overweight	11 (15.5)	30 (20.4)	41 (18.8)	
Obesity	5 (7.0)	13 (8.8)	18 (8.3)	

* – Body Mass Index (BMI) was calculated using the ratio of weight (kilograms) to the square of height (meters) kg/m^2^. Underweight (<18.5). Normal weight (≥18.5–24.9 kg/m^2^), overweight (>24.9–29.9 kg/m^2^), obesity (>29.9 kg/m^2^) (WHO, 2000).

### Maternal health during pregnancy among mothers of LBW newborns and normal-birth-weight newborns

To assess maternal health, the following factors were taken into consideration: supplementation of iron, calcium, and vitamin D, weight gain during pregnancy, prenatal visit frequency, pregnancy-related anemia, utilization of other medications, dietary intake, sleep quality, and various health conditions (Table 2). The results indicated that daily supplementation of iron, calcium, and vitamin D was significantly more prevalent among mothers of normal-weight newborns (85.8%, n=127) compared to mothers of low-birth-weight newborns (30.0%, n=41) (p<0.001). In terms of weight gain during pregnancy, mothers of low birth-weight newborns had a higher prevalence of gestational weight gain of 6–10 kg (31.9%, n=23) compared to mothers of normal-weight newborns (23.6%, n=36). Conversely, mothers of normal-weight newborns had a higher prevalence of gestational weight gain exceeding 10 kg (76.3%, n=113) compared to mothers of low birth-weight newborns (68.1%, n=49) (p=0.001) (Table 2).

**Table 2:** Maternal physical and medical health during pregnancy (n=220).

Variables	Case (72) n (%)	Control (148) n (%)	Total	P-value
**Iron, calcium and vitamin D use**				
Yes, not daily	42 (58.3)	21 (14.2)	63 (28.6)	**<0.001**
Yes, daily	30 (41.7)	127 (85.8)	157 (71.4)	
**Maternal weight gain (kg)**				
6–10	23 (31.9)	35 (23.6)	58 (26.4)	**0.001**
11–16	30 (41.7)	98 (66.2)	128 (58.2)	
>16	19 (26.4)	15 (10.1)	34 (15.4)	
**Prenatal visits**				
1–3	4 (5.6)	3 (2.0)	7 (3.2)	**<0.001**
4–7	28 (38.9)	25 (16.9)	53 (24.1)	
>7	40 (55.6)	120 (81.1)	160 (72.7)	
**Maternal anemia**				
Yes	45 (62.5)	20 (13.5)	65 (29.5)	**<0.001**
No	27 (37.5)	128 (86.5)	155 (70.5)	
**Regularly medications**				
Yes	4 (5.6)	4 (2.7)	8 (3.6)	0.289
No	68 (94.4)	144 (97.3)	212 (96.4)	
**Healthy food intake**				
Yes	19 (26.4)	116 (78.4)	135 (61.4)	<0.001
No	53 (73.6)	32 (21.6)	85 (38.6)	
**Sleeping well**				
Yes	36 (50.0)	80 (54.1)	116 (52.7)	0.572
No	36 (50.0)	68 (45.9)	104 (47.3)	
**Rupture of Membrane**				
Yes	59 (83.1)	136 (91.9)	24 (11.0)	0.051
No	12 (16.9)	12 (8.1)	195 (89.0)	
**Diabetes**				
Yes	4 (5.6)	7 (4.7)	11 (5.0)	0.792
No	68 (94.4)	141 (95.3)	209 (95.0)	
**Recurrent Urinary Tract Infections (UTI)**				
Yes	16 (22.5)	19 (12.8)	35 (16.0)	0.067
No	55 (77.5)	129 (87.2)	184 (84.0)	
**Hypertension**				
Yes	11 (15.3)	8 (5.4)	19 (8.6)	**0.014**
No	61 (84.7)	140 (94.6)	201 (91.4)	
**Asthma**				
Yes	4 (5.6)	3 (2.0)	7 (3.2)	0.156
No	67 (94.4)	145 (98.0)	212 (96.8)	
**Rheumatic disease**				
Yes	3 (4.2)	8 (5.4)	11 (5.0)	0.708
No	68 (95.8)	140 (94.6)	208 (95.0)	
**Systemic lupus erythematosus**				
Yes	3 (4.2)	3 (2.0)	6 (2.7)	0.361
No	69 (95.8)	145 (98.0)	214 (97.3)	
**Other medical condition**				
Yes	2 (2.8)	9 (6.1)	11 (5.0)	**0.291**
No	70 (97.2)	139 (93.9)	209 (95.0)	

The number of prenatal visits was negatively associated with LBW newborns (p<0.001). Maternal anemia was higher among mothers of LBW newborns (62.5%, n=45) compared to mothers of normal-birth-weight newborns (13.5%, n=20) (p<0.001). Women who had access to healthy food during pregnancy were higher among mothers of normal-weight newborns (78.4%, n=116) than among mothers of LBW newborns (26.4%, n=16) (p<0.001) (Table 2). Additionally, hypertension was more prevalent among women who had low birth weight newborns (15.3%, n=11) compared to women who had normal-birth-weight newborns (5.4%, n=8) (p=0.014) (Table 2).

### Obstetric outcomes among mothers of LBW and normal- birth weight newborns

A chi-square test (Fisher's exact test) was performed to analyze the distribution of categorical obstetric outcomes variables between the two groups, including parity, neonatal sex, pregnancy planning, previous miscarriage, gestational age, type of pregnancy, delivery mode, and previous LBW newborn (Table 3). 51.4% (n=37) of the LBW newborns were female, while 48.6% (n=35) were male. In contrast, the control group comprised 68.3% (n=99) males and 31.7% (n=46) females (Table 3). The percentage of women who planned their pregnancy in the control group (87.8%, n=130) was significantly higher (p=0.001) than in the case group (62.5%, n=45) (Table 3). Moreover, LBW newborns were significantly more likely to be born through vaginal delivery (63.9%, n=46) compared to cesarean section (36.1%, n=26) (p<0.004) (Table 3). Additionally, the number of mothers with a history of LBW was significantly higher in the case group (33.3%, n=24) compared to the control group (7.4%, n=11) (p<0.001) (Table 3).

**Table 3:** Maternal obstetric health (n=220).

Variables	Case (72) n (%)	Control (148) n (%)	Total	P-value
**Parity**				
Primigravida	30 (41.7)	58 (39.2)	88 (40.0)	0.725
Multi-gravida	42 (58.3)	90 (60.8)	132 (60.0)	
**Neonatal gender**				
Boy	35 (48.6)	99 (68.3)	134 (61.8)	**0.005**
Girl	37 (51.4)	46 (31.7)	83 (38.2)	
**Planning for pregnancy**				
Yes	45 (62.5)	130 (87.8)	175 (79.5)	**<0.001**
No	27 (37.5)	18 (12.2)	45 (20.5)	
**Previous miscarriage**				
Yes	10 (13.9)	11 (7.4)	21 (9.5)	0.126
No	62 (86.1)	137 (92.6)	199 (90.5)	
**Type of pregnancy**				
Induced	68 (94.4)	128 (87.1)	196 (89.5)	0.095
Spontaneous	4 (5.6)	19 (12.9)	23 (10.5)	
**Gestational age (week)**				
Preterm (37–38)	10 (13.9)	11 (7.4)	21 (9.5)	0.126
Full-term (38–42)	62 (86.1)	137 (92.6)	199 (90.5)	
**Mode of delivery**				
Cesarean section	26 (36.1)	27 (18.2)	53 (24.1)	**0.004**
Vaginal delivery	46 (63.9)	121 (81.8)	167 (75.9)	
**Previous LBW newborn**				
Yes	24 (33.3)	11 (7.4)	35 (15.9)	**<0.001**
No	48 (66.7)	137 (92.6)	185 (84.1)	

### The relationship between COVID-19 infection, socioeconomic impact, and LBW

The infection rate among the case group was significantly higher compared to the control group, with 43.1% (n=31) and 9.5% (n=14), respectively (p<0.001). Hospitalization due to COVID-19 was also more frequent in the case group (16.7%, n=12) compared to the control group (1.4%, n=2) (p<0.001). The impact of the COVID-19 pandemic on employment was greater in the case group, with 83.3% of mothers of LBW newborns losing their jobs compared to only 4.7% in the control group (p<0.004). Additionally, 91.7% of mothers in the case group reported a financial impact from the pandemic, compared to 25.0% in the control group (p<0.001). Antenatal care schedules, monthly medications, iron, calcium, vitamin D supplementation, and food access were also more frequently disrupted in the case group than in the control group due to the pandemic (p<0.001) (Table 4).

**Table 4:** COVID-19 infection and socio-economic deterioration (n=220).

Variables	Case (72) n (%)	Control (148) n (%)	Total	P-value
**COVID-19 infection**				
Yes	31 (43.1)	14 (9.5)	45 (20.5)	**<0.001**
No	41 (56.9)	134 (90.5)	175 (79.5)	
**Hospital admission**				
Yes	12 (16.7)	2 (1.4)	14 (6.4)	**<0.001**
No	60 (83.3)	146 (98.6)	206 (93.6)	
**Worrying about having COVID-19**				
Yes	68 (94.4)	135 (91.2)	203 (92.3)	0.400
No	4 (5.6)	13 (8.8)	17 (7.7)	
**Losing work**				
Yes	55 (83.3)	7 (4.7)	62 (29.0)	**0.004**
No	11 (16.7)	141 (95.3)	152 (71.0)	
**Family members losing work**				
Yes	58 (80.6)	23 (15.5)	81 (36.8)	**<0.001**
No	14 (19.4)	125 (84.5)	139 (63.2)	
**Economic ability**				
Yes	66 (91.7)	37 (25.0)	103 (46.8)	**<0.001**
No	6 (8.3)	111 (75.0)	117 (53.2)	
**ANC visits schedule**				
Yes	59 (83.1)	65 (43.9)	124 (56.6)	**<0.001**
No	12 (16.9)	83 (56.1)	95 (43.4)	
**Monthly medication**				
Yes	50 (69.4)	40 (27.0)	90 (40.9)	**<0.001**
No	22 (30.6)	108 (73.0)	130 (59.1)	
**Iron, calcium and vitamin D supplements**				
Yes	59 (81.9)	57 (38.5)	116 (52.7)	**<0.001**
No	13 (18.1)	91 (61.5)	104 (47.3)	
**Food supply**				
Yes	60 (83.3)	15 (10.1)	75 (34.1)	**<0.001**
No	12 (16.7)	133 (89.9)	145 (65.9)	

### Predictors of LBW

Seven out of 24 predictors were statistically significant, including monthly income, daily intake of iron, calcium, and vitamin D supplements, passive smoking, maternal anemia, gestational age, COVID-19 infection, and the impact of the COVID-19 pandemic on food supply (Table 5). The logistic regression analysis showed that a monthly income of 250–400 JD was associated with a 12.047-fold increase in the likelihood of having an LBW newborn compared to mothers with a monthly income of over 700 JD while controlling for other variables in the model [95% CI:1.680–86.401].

**Table 5:** Binary logistic regression analysis of LBW-related risk factors.

Variable (in the final model ^a^)	B	Exp (B)	Odds Ratio (95% CI)
**Monthly income**			
≤400	2.489	12.047*	(1.680–86.401)
401-550	.719	2.052	(0.290–14.506)
550-700	1.149	3.154	(0.524–18.973)
>700	-	-	-
**Passive smoking (yes)**	2.405	11.078*	(2.570–47.741)
**Iron, calcium and vitamin D supplements (daily)**	-1.857	0.156*	(0.040–0.610)
**Maternal anemia (yes)**	1.607	4.986*	(1.287–19.318)
**Gestational age**			
Preterm	2.149	8.577*	(1.004–73.301)
Full-term	-	-	-
**COVID-19 infection (yes)**	2.545	12.745*	(2.510–64.719)
**Food supply affected by the COVID-19 pandemic (yes)**	2.880	17.806*	(2.196–144.343)

* – Statistically significant at P<0.05; ^a^ – Variables in the first step were all variables with a p-value of ≤0.2 in the chi-square test.

Mothers exposed to passive smoking during pregnancy were more likely to have an LBW newborn (OR=2.405; 95% CI: 2.570–47.741). Daily supplementation intake increased the odds of having a normal-weight newborn by 6.41 times compared to those who did not take daily supplements (95% CI: (0.040–0.610). Pregnant women with anemia were more likely to have a low birth weight newborn compared to those without anemia (OR=4.986; 95%CI: 1.287–19.318). Preterm delivery had 8.577 times higher odds of a low birth weight compared to full-term delivery (95% CI: 1.004–73.301). Mothers infected with COVID-19 had 12.75 times higher odds of having a low birth weight newborn (95% CI: 2.510–64.719). Moreover, mothers whose food supply was affected by the COVID-19 pandemic had 17.806-fold higher odds of having a low birth weight newborn than those whose food supply was not impacted (95% CI: 4.93–27.78).

## DISCUSSION

This study aimed to investigate the relationship between maternal health factors and COVID-19 infection with low birth weight (LBW) in Jordan. Our results showed that a lower monthly income was linked to a higher likelihood of having an LBW newborn. Specifically, more mothers of LBW newborns had incomes below 400 JD in comparison to mothers of normal birth weight babies. The results are in line with other studies suggesting that pregnant women with low incomes may not be able to receive adequate nutrition and health care [9–12]. Pregnant women exposed to secondhand smoke during pregnancy had an increased risk of delivering low birth weight newborns, which is supported by several studies that link passive smoking to LBW [13–17]. The harmful effects of secondhand smoke on fetal growth and development can be attributed to the chemicals exhaled by smokers. Carbon monoxide, a component of secondhand smoke, reduces oxygen delivery to the fetus by forming carboxyhemoglobin and causing vasoconstriction through nicotine [18, 19].

Our findings indicate that a higher proportion of mothers of LBW newborns live with their extended family. This is probably due to a decrease in monthly income per person, which is associated with poorer diet quality during pregnancy [20]. Furthermore, the intake of iron, calcium, and vitamin D supplements during pregnancy was linked to a decreased probability of having a low birth weight newborn, corresponding to other studies [21–23]. The underlying mechanisms behind this association may include improved gestational weight gain and prevention of anemia, which can positively impact both the mother and the fetus [21, 24].

We found no significant association between LBW and the mother's body mass index (BMI) (p=0.753), which contradicts previous findings [25, 26] that obesity increases the risk of LBW. However, the study did reveal an association between gestational weight gain and LBW risk. Pregnant women with a gestational weight gain of 6–10 kg had a higher risk of delivering LBW newborns, while those with a gestational weight gain of 10–16 kg had a lower risk. These findings are consistent with previous studies [5, 27–29]. For example, Zhao *et al*. [29] found that pregnant women with a gestational weight gain below the recommended range specified by the American Institute of Medicine (11.3–15.9 kg for those with normal pre-BMI) were at a higher risk of delivering low birth weight newborns, compared to women who had a gestational weight gain within this range.

The number of prenatal visits was also found to have a negative association with LBW outcomes. These findings are consistent with previous research, which has demonstrated that pregnant women who did not attend antenatal care at least four times are more susceptible to LBW outcomes [30–32]. Additionally, there was a positive correlation between maternal anemia and LBW outcomes. This association has been previously established in a multitude of studies [33, 34] and was reinforced by the results of a systematic review and meta-analysis, which concluded that maternal anemia is positively associated with LBW outcomes [33]. Access to healthy food intake during pregnancy was a significant risk factor for LBW, with women without access being 2.88 times more likely to have an LBW newborn than those with access. These findings are in agreement with several studies [35–37]. Abubakari and Jahn [36] documented that a balanced dietary intake during pregnancy was positively correlated with a reduced risk of LBW.

A higher prevalence of hypertension was observed among mothers of LBW newborns, a correlation substantiated by the findings of Liu *et al*. [38] and Rahman *et al*. [39]. Furthermore, female neonates were identified as being more susceptible to LBW, a result that concurs with the findings of Afaya *et al*. [40], Agorinya *et al*. [41], and Manyeh *et al*. [42]. Afaya *et al*. [40] reported that female neonates had 64% higher odds of LBW than male neonates.

Our results showed that the risk for LBW decreased significantly when the pregnancy was planned, supporting the result of other studies where unplanned pregnancies increased the risk of low birth weight by 24% compared to planned pregnancies [43, 44]. A higher rate of LBW was observed within cesarean deliveries compared to vaginal deliveries. The results align with Chen *et al*. [45] and Taha *et al*. [46]. Chen *et al*. [45] found that the rate of cesarean section of LBW was 1.24 times higher than those of normal birth weight newborns. During the last ten years, the rate of cesarean deliveries in Jordan has increased [47–49]. More than fifty percent of women delivered by C-sections before 39 weeks of gestation, which is associated with a higher risk of neonatal complications [49].

Women with a history of LBW were significantly more likely to have recurrent LBW newborns, corresponding with other studies [50–53]. For example, Mvunta *et al*. [52] reported that women with a history of LBW were more likely to have recurrent LBW in late pregnancy compared to those who had a previous normal birth weight baby.

This study also demonstrated that LBW was significantly associated with COVID-19 infection, hospital admission, losing work, and financial ability affected by the COVID-19 pandemic. Previous studies have shown that the COVID-19 pandemic adversely affects pregnant women and their newborns [34, 54, 55]. The COVID-19 outbreak was considered a major stress that may have negatively affected intrauterine development and increased preterm birth rates as well as low birth weight rates [54]. Previous studies have shown that the COVID-19 pandemic has negatively impacted pregnant women and newborns, potentially increasing stress levels, anxiety, and depression and leading to higher rates of preterm birth and LBW [56]. As a result, the preterm birth rate may rise, and intrauterine growth restriction, particularly low birth weight, may become more common.

## CONCLUSION

The present study aimed to identify the risk factors associated with low birth weight (LBW) among pregnant women in Jordan. Results showed that several factors were significantly associated with LBW, including monthly income, daily intake of iron, calcium, and vitamin D supplements, exposure to passive smoking, maternal anemia, gestation age, COVID-19 infection, and disruptions in food supply and financial ability due to the COVID-19 pandemic.

The findings of this study highlight the need for comprehensive educational programs for pregnant women that focus on prenatal care, proper nutrition, and supplement intake. Regular screening tests, including those for LBW, should also be a priority to prevent and manage this serious public health issue.

It is important to note that these findings should be further validated through additional research in various hospital sectors across Jordan. Such efforts will contribute to a better understanding of the prevalence and risk factors of LBW, ultimately leading to improved maternal and fetal outcomes.

## Personal thanks

The authors would like to thank all participants in this study and the Research Faculty at Jordan University of Science and Technology.
